# Supplementary material for: Therapeutic Effects of Inhibition of Sphingosine-1-Phosphate Signaling in HIF-2α Inhibitor-Resistant Clear Cell Renal Cell Carcinoma
Source: Cancers (Basel). 2021 Sep 25;13(19):4801. doi: 10.3390/cancers13194801 (PMC8508537; doi:10.3390/cancers13194801)

Supplementary Figure S1

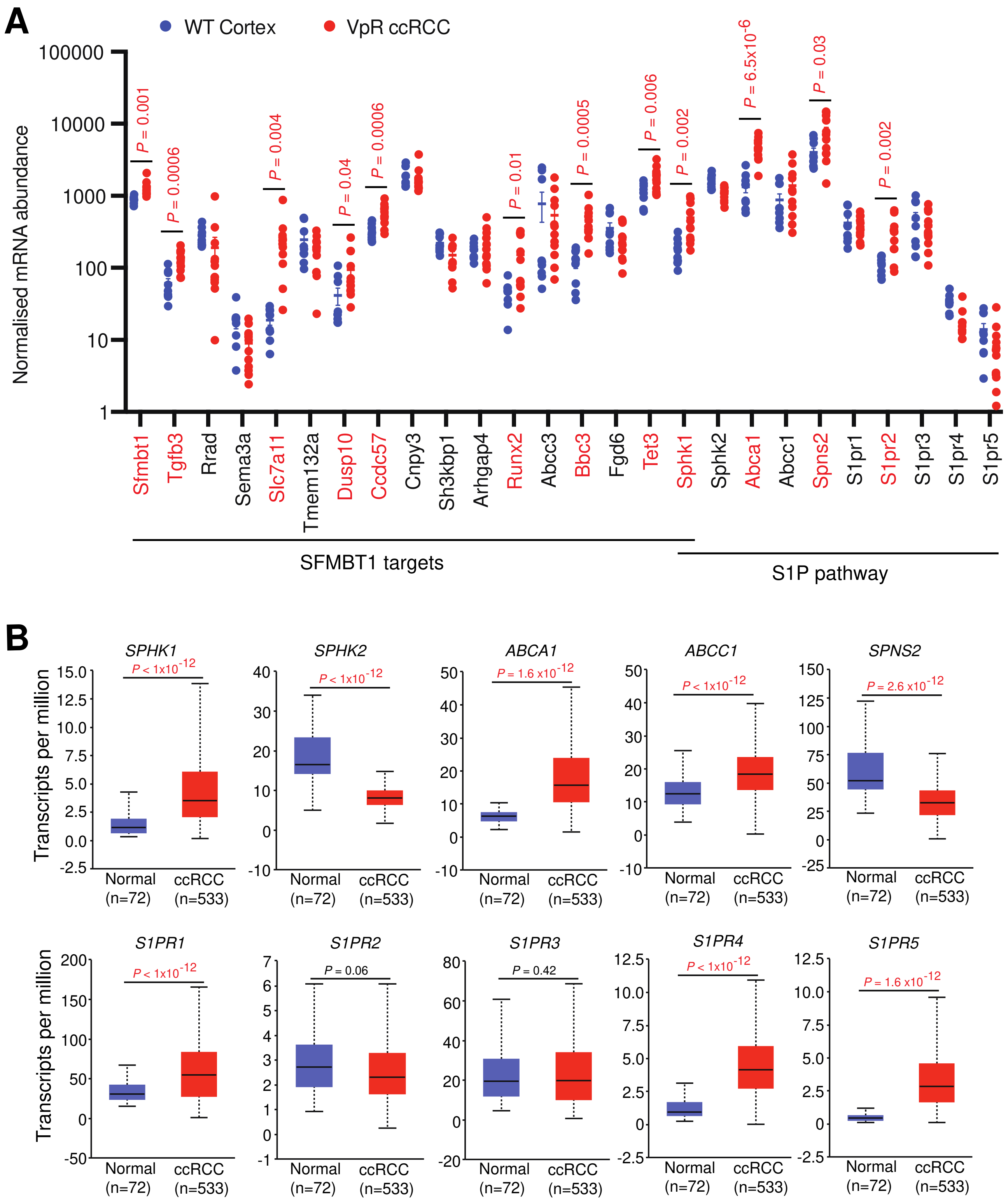

Supplementary Figure S2

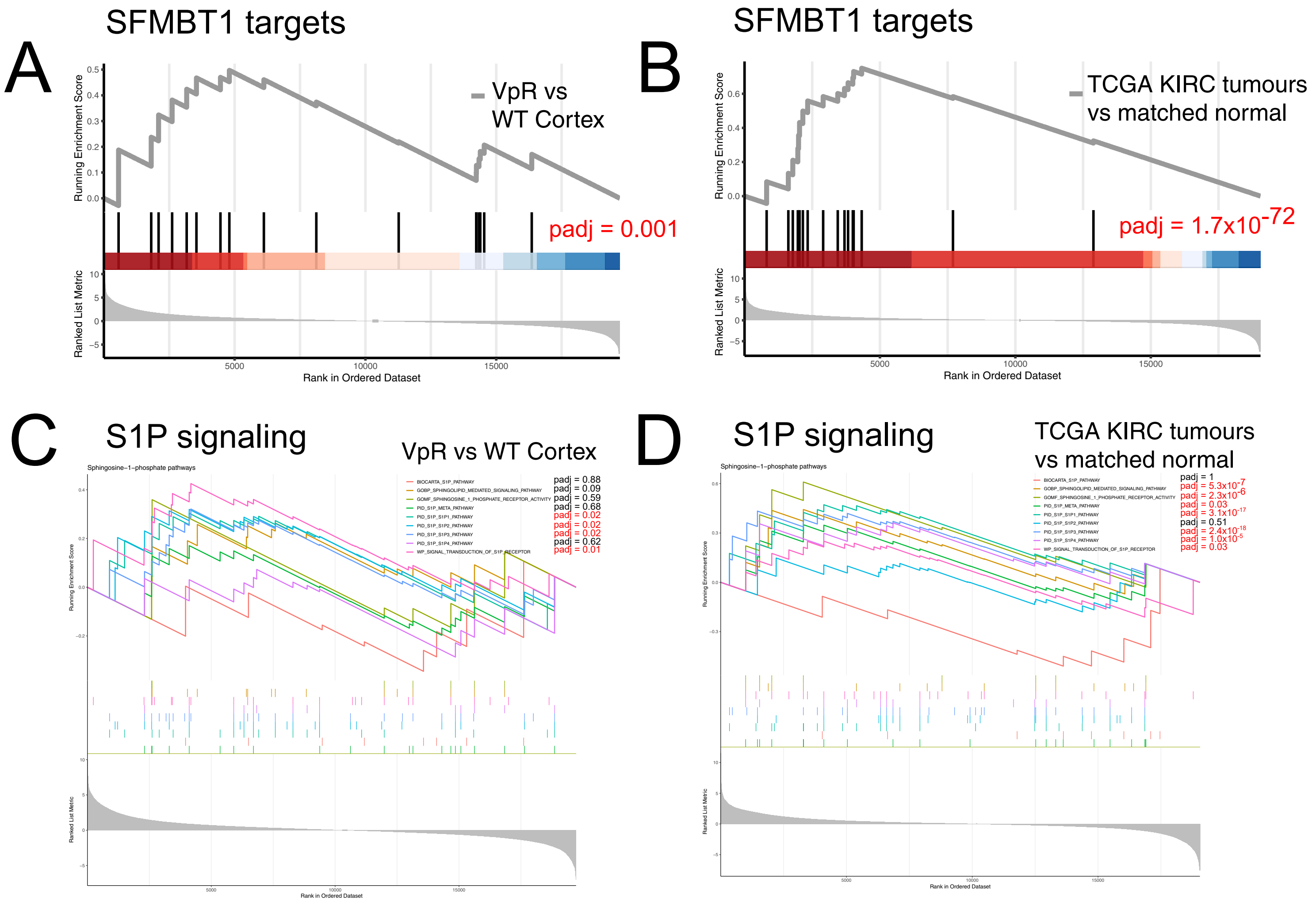

Supplementary Figure 3

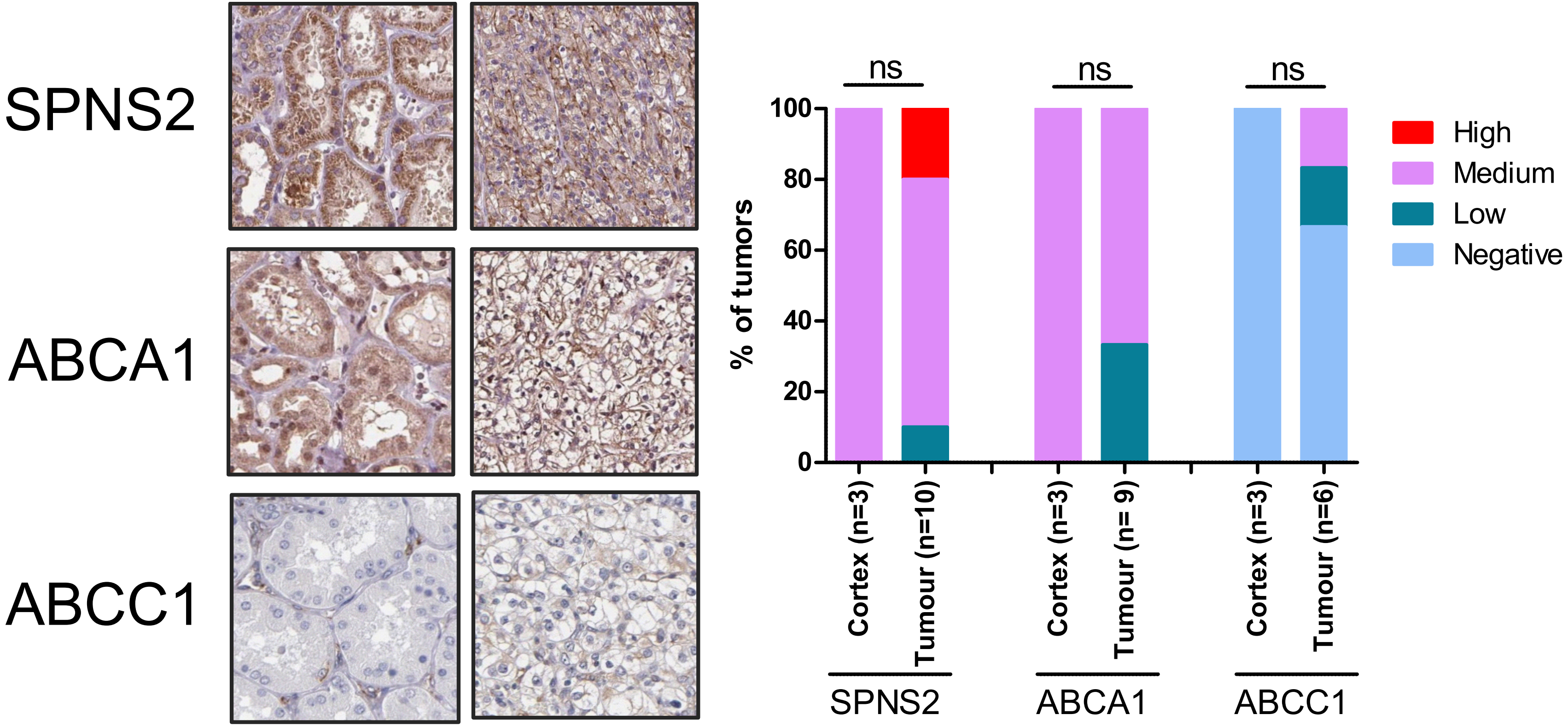

Supplementary Figure S4

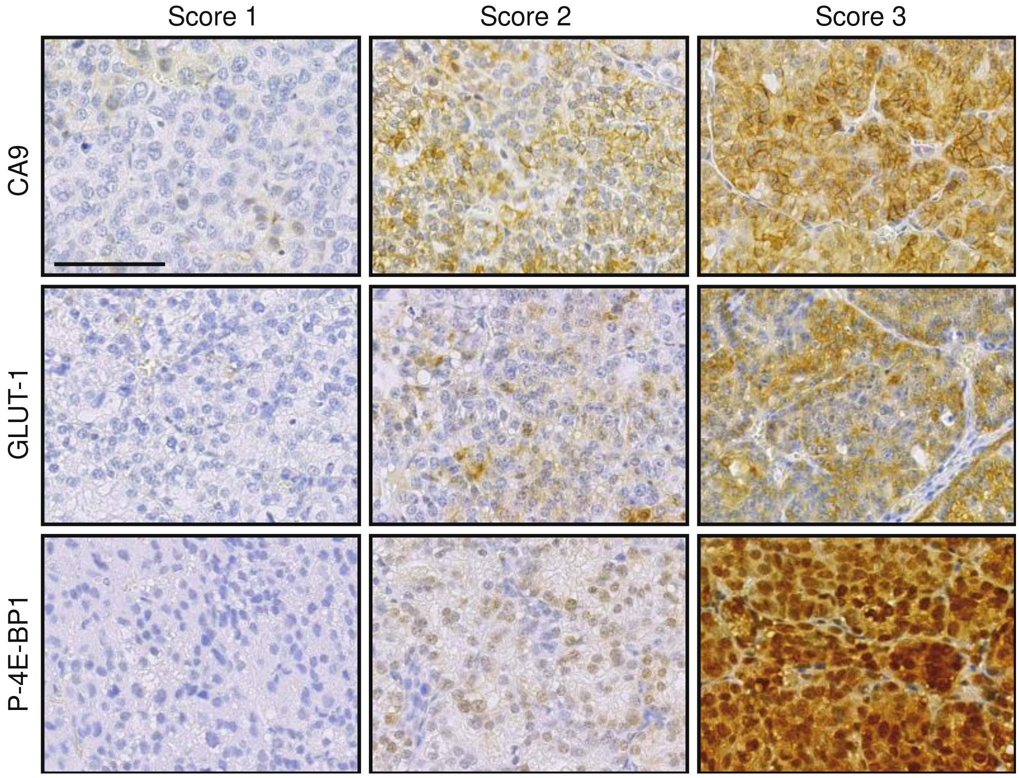

Supplement: Supplementary file 1 [file cancers-13-04801-s001.zip › suppl/Supplementary_Figures_revision_final_vector_without_legend.pdf]
