# Supplementary material for: Therapeutic Effects of Inhibition of Sphingosine-1-Phosphate Signaling in HIF-2α Inhibitor-Resistant Clear Cell Renal Cell Carcinoma
Source: Cancers (Basel). 2021 Sep 25;13(19):4801. doi: 10.3390/cancers13194801 (PMC8508537; doi:10.3390/cancers13194801)
Supplement: Supplementary file 1 [file cancers-13-04801-s001.zip › suppl/Supplementary_Figures_revision_with_legend_final.pdf]

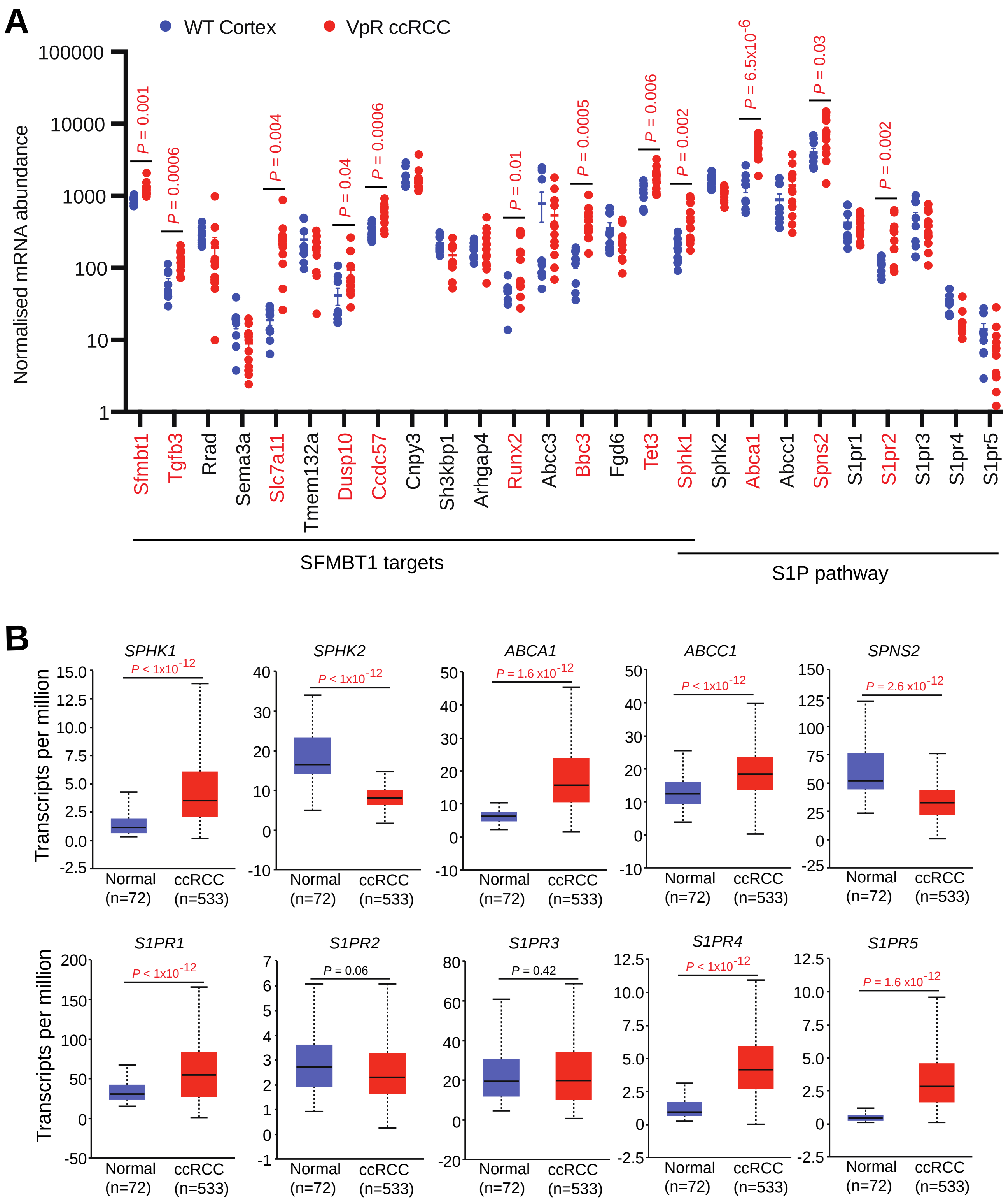

Supplementary Figure S1. Expression of SFMBT1 target and S1P signaling pathway genes in mouse and human ccRCC : (A) Distributions and Mean ± SEM expression levels of the indicated genes in WT Cortex (n=9) and VpR (n=12), ccRCC tumors derived from RNA sequencing analyses. Two-sided *P* values were calculated by Student's t-test. (**B**) mRNA abundance (log_2 transformed, normalised, RNAseq v2 RSEM) of the indicated genes in normal cortex and ccRCC tumors in the TGCA KIRC dataset. *P* values are derived from two-sided Student's t-test.

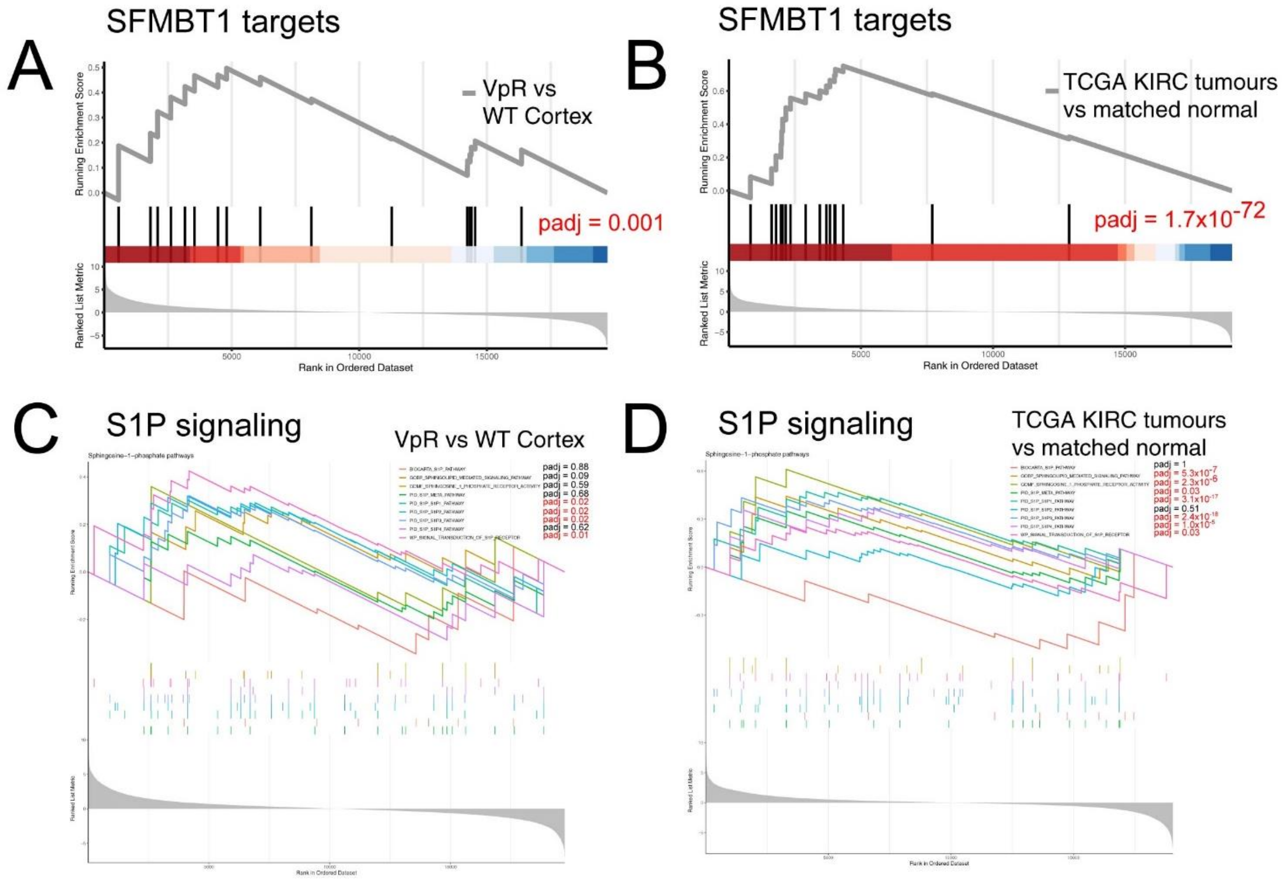

Supplementary Figure S2. Gene-set enrichment plots: (A-B) Gene-set enrichment plots of SFMBT1 targets in mouse ccRCC compared to normal cortex **(A)** or human (TCGA KIRC) ccRCC compared to matched normal tissue **(B)**. **(C-D)** Gene-set enrichment plots of the nine indicated S1P-signalling gene-sets (derived from the MSigDB platform) in mouse ccRCC compared to normal cortex **(C)** or human ccRCC (TCGA KIRC) compared to matched normal tissue **(D)**. Log2 fold changes between tumour and normal samples were calculated and GAGE (Generally Applicable Gene-Set Analysis) was performed on the respective, transformed counts. *P*-values were derived from Student's t test and were adjusted via the Benjamini-Hochberg correction. Pathways were considered significant with an adjusted *P*- value < 0.05. For the visualization, enrichment plots were generated by ranking all genes for each dataset based on the log2FC.

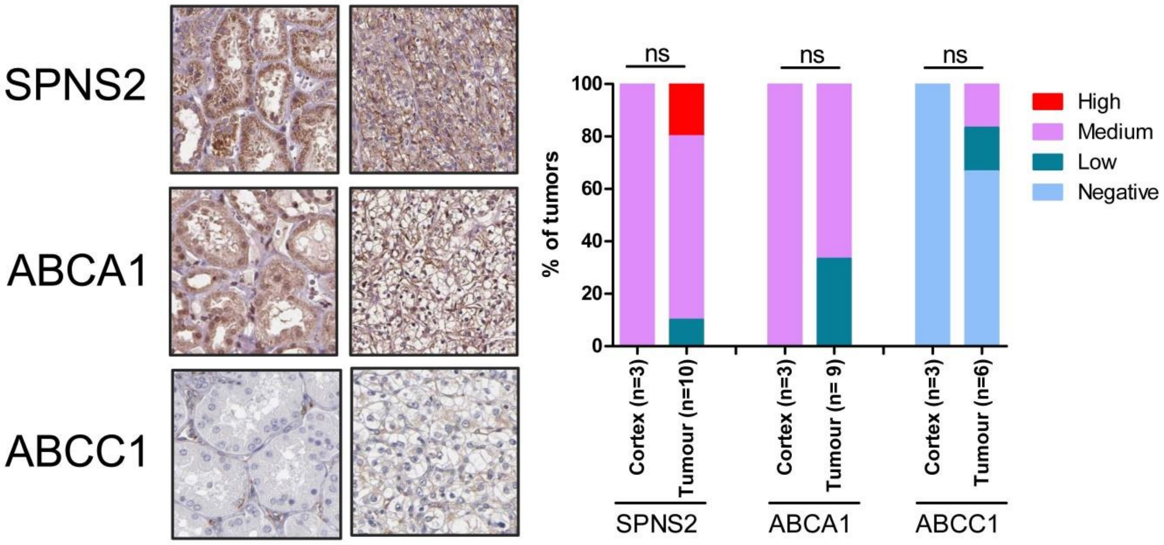

Supplementary Figure S3. Expression of S1P-transporters in human ccRCC: Representative pictures of IHC stainings of the indicated S1P-transporters in human ccRCC and normal kidney cortex derived from the Human Protein Atlas platform. Bar-plot showing expression levels of the S1P-transporters in human ccRCC and normal kidney cortex. Statistical significance was assessed by the two-sided Student's t-test; ns = non-significant.

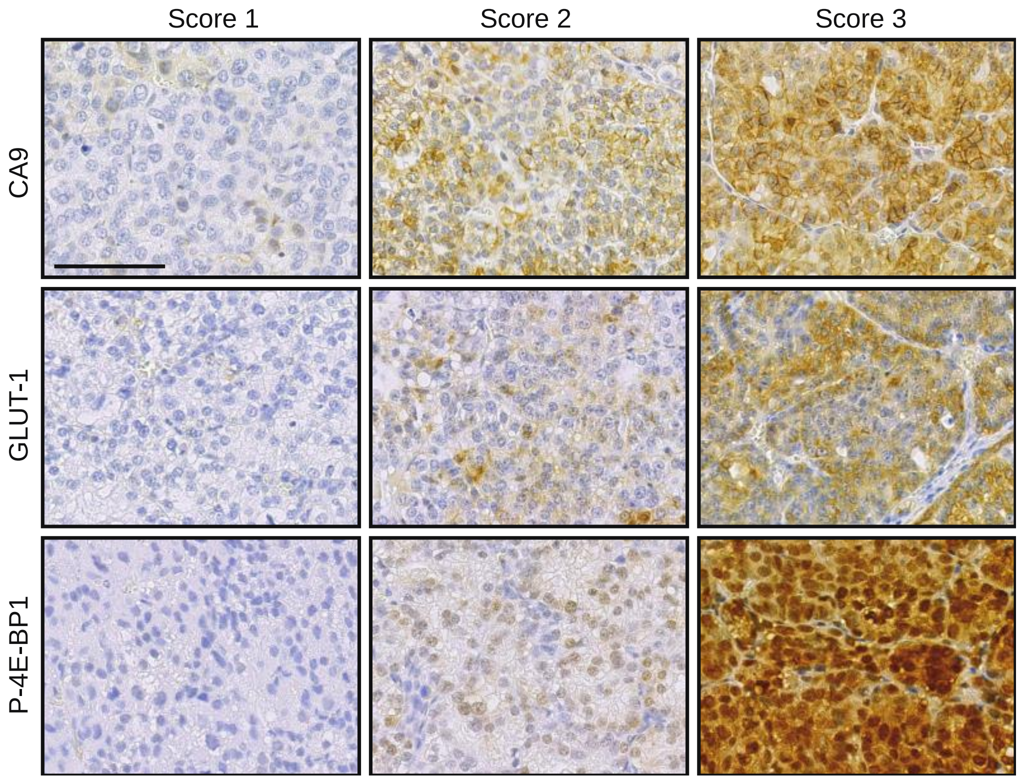

Supplementary Figure S4. Examples of scoring of immunohistochemical stainings for the indicated antibodies. Scale bar = 100mm.
