# Supplementary figures and images for: Therapeutic Effects of Inhibition of Sphingosine-1-Phosphate Signaling in HIF-2α Inhibitor-Resistant Clear Cell Renal Cell Carcinoma
Source: Cancers (Basel). 2021 Sep 25;13(19):4801. doi: 10.3390/cancers13194801 (PMC8508537; doi:10.3390/cancers13194801)

Figure 4E

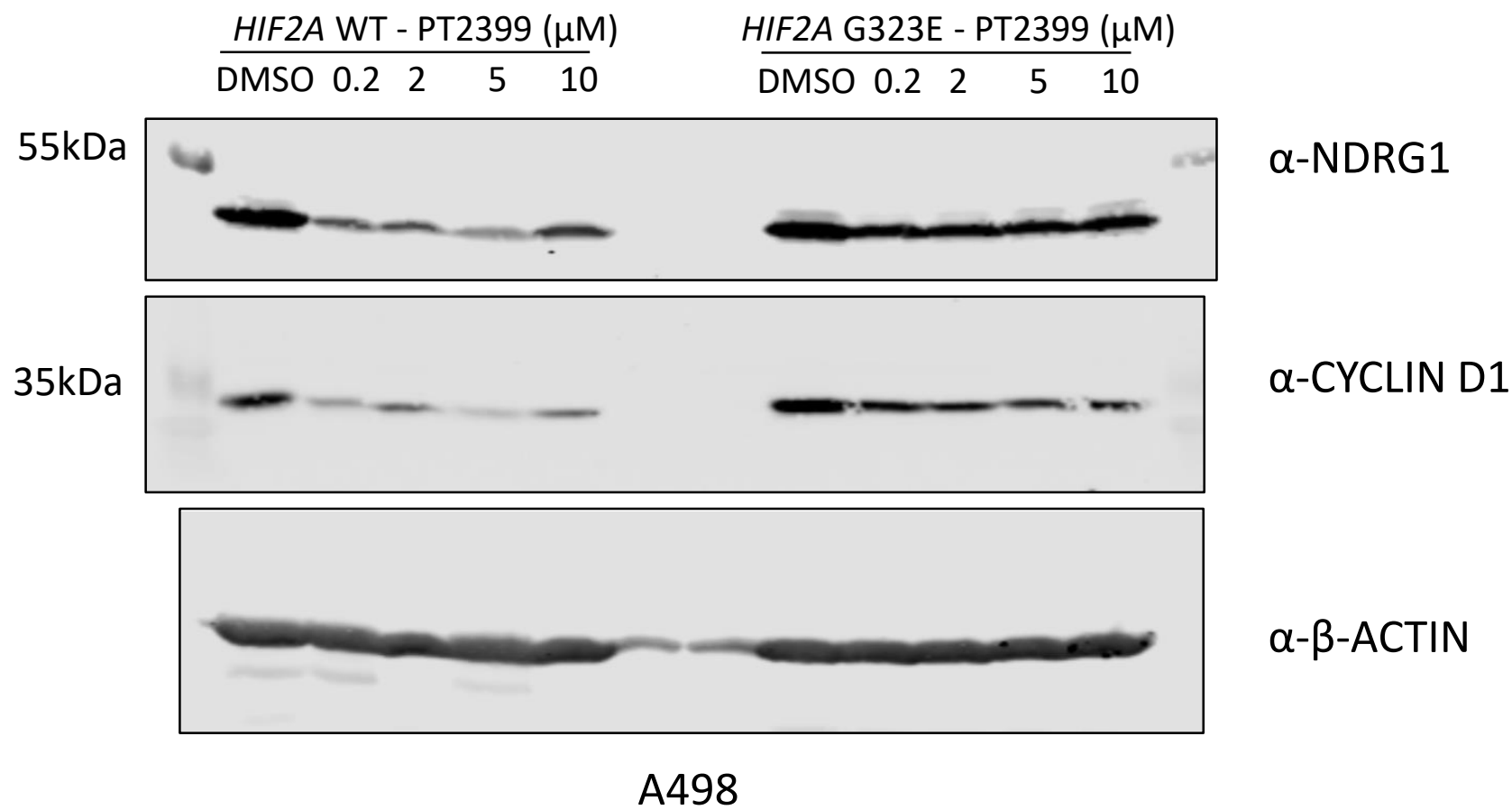

Figure 5G

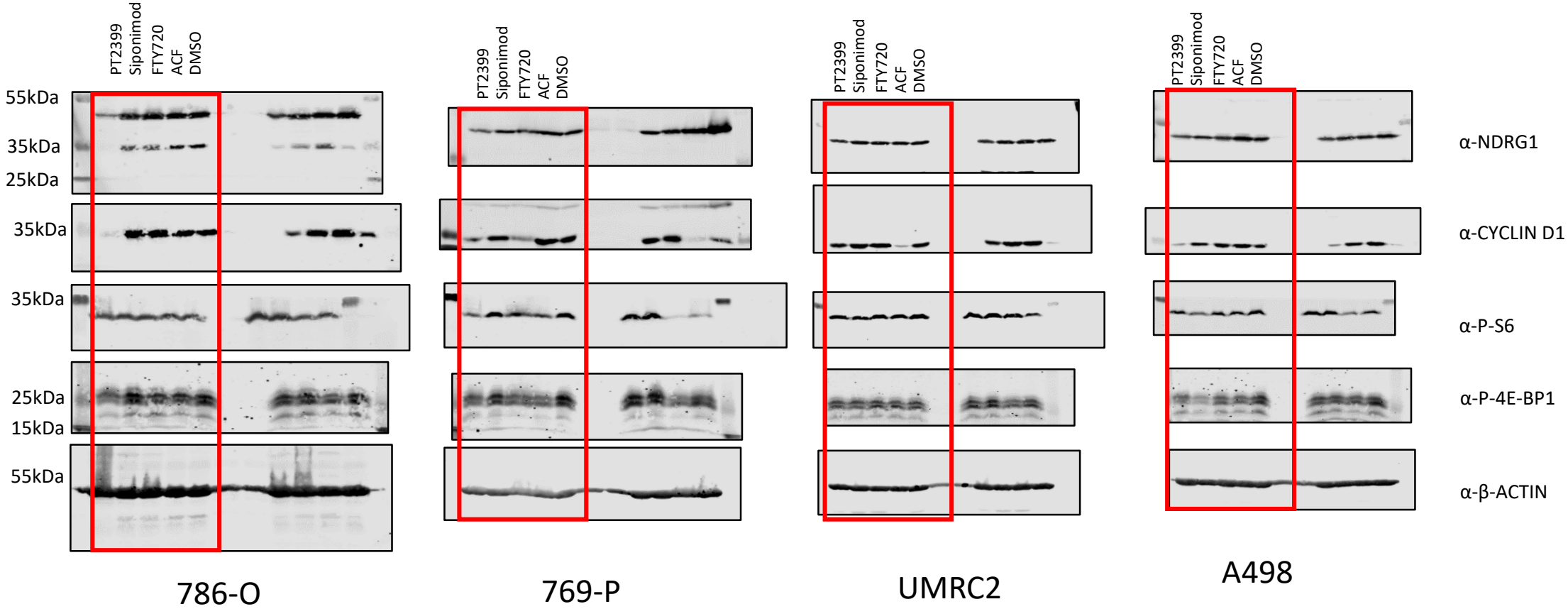

Supplement: Supplementary file 1 [file cancers-13-04801-s001.zip › suppl/cancers-1398854-original image.pdf]
